# A Cross-Cultural Comparative Study on Italian and American University Students’ Psychological Symptoms and the Predicting Role of Personality Traits

**DOI:** 10.3390/ejihpe15090175

**Published:** 2025-08-29

**Authors:** Sara Guidotti, Gabriella Coscioni, Carlo Pruneti

**Affiliations:** 1 Clinical Psychology, Clinical Psychophysiology, and Clinical Neuropsychology Laboratories, Department of Medicine and Surgery, University of Parma, 43126 Parma, Italy; carlo.pruneti@unipr.it; 2 Department of Psychology and Neuroscience, Boston College, Chestnut Hill, MA 02467, USA; gcoscioni@journeyaba.com

**Keywords:** mental health, personality, cross-cultural, university students, college students

## Abstract

(1) Background: This study aimed to compare psychological symptoms between Italian university students and American college students, considering both external (e.g., nationality) and internal variables (e.g., gender, age, and personality traits) potentially associated with mental health status. (2) Methods: A total of 201 Italian students from the University of Parma and 214 American students from Boston College were recruited. Participants completed the Symptom Questionnaire (SQ) and the 16 Personality Factors Questionnaire (16PF). Group comparisons were conducted on socio-demographic and psychological variables using chi-square and independent samples *t*-tests. Subsequently, hierarchical linear regression analyses were performed separately for each sample to identify personality traits that predict psychological symptoms, while controlling for gender and age. (3) Results: Both samples scored above the clinical cut-off on all SQ symptom scales. American students reported significantly higher levels of depression and hostility. Across both groups, psychological distress was primarily predicted by high tension and low emotional stability, with personality traits explaining a greater proportion of variance than nationality. Traits related to social interaction and emotional regulation also emerged as significant predictors. (4) Conclusions: Identifying personality profiles that are more vulnerable to psychological symptoms may support the development of early identification strategies and targeted prevention programs in university settings.

## 1. Introduction

Mental health among university students has become a growing global public health concern. The transition into higher education often coincides with a convergence of internal and external stressors, increasing vulnerability to psychological symptoms such as anxiety, depression, and somatization. These stressors include academic demands, financial pressures, interpersonal changes, and family expectations, which challenge students’ coping abilities in diverse sociocultural contexts (Acharya et al., 2018; Lyrakos, 2012). Among them, academic stressors, such as exams, performance anxiety, and workload, are consistently reported as key contributors to psychological distress across populations (Saleh et al., 2017; Sussman & Arnett, 2014).

In recent years, the COVID-19 pandemic has intensified these vulnerabilities by introducing a prolonged, global stressor that further disrupted academic life and interpersonal functioning (Baltà-Salvador et al., 2021; Son et al., 2020). Students faced increased isolation, uncertainty, and challenges with online education. These pandemic-related pressures compounded existing stressors, such as performance demands and social disconnection, resulting in elevated levels of anxiety, depression, and somatic complaints (Hoyt et al., 2021; Son et al., 2020; Yang et al., 2021).

However, the effects of such stressors are shaped by the broader cultural and institutional context. Previous research highlights that nationality, educational structures, and cultural expectations significantly influence students’ access to support systems, coping strategies, and psychological resilience (Lyrakos, 2012). For instance, the United States and Italy differ markedly in their higher education systems, with implications for stress exposure and appraisal. American college students typically begin university at age 18 and follow a four-year program structured around continuous assessment (e.g., essays, midterms, GPA-based grading). This model promotes steady academic engagement but may lead to chronic, low-grade stress. Conversely, Italian students typically begin their studies at 19 and complete degrees over three or five years, with final grades often determined by a single oral exam per course. Exams can be repeated, which may reduce pressure in the short term but contributes to long-term academic uncertainty and prolonged study periods.

In addition to these structural differences, internal psychological factors strongly contribute to students’ stress responses. Gender is a frequently cited factor, with female students generally reporting higher levels of stress, anxiety, and depression than males (Hoyt et al., 2021; Fogle & Pettijohn, 2013; Garett et al., 2017). While age can influence psychological vulnerability, especially during developmental transitions, its effect appears less consistent compared to other factors such as emotional maturity and autonomy (Saleh et al., 2017; Sussman & Arnett, 2014). In this regard, personality traits are increasingly recognized as robust internal predictors of mental health. Low emotional stability (i.e., high neuroticism), for instance, is closely associated with elevated levels of perceived stress, anxiety, and depression (Fogle & Pettijohn, 2013; Kural & Özyurt, 2021). Other traits, such as conscientiousness, extraversion, openness, and self-control, can shape how individuals respond to academic and social demands (Karyotaki et al., 2020). These temperamental characteristics not only modulate stress perception but also influence the emergence of psychological symptoms (Guidotti et al., 2022).

Moreover, certain personality configurations proved to have indirect effects through mediating variables. Specifically, perfectionism and low emotional stability may increase the risk of anxiety and somatization through the expression of Type A behavior patterns characterized by competitiveness, impatience, and urgency (Guidotti et al., 2024a). Similarly, the combination of introversion, alexithymia, and poor emotional regulation has been linked to hostility and even suicidal ideation among students, highlighting the importance of emotional awareness and interpersonal functioning in mental health (Guidotti et al., 2024b).

Alongside internal factors, external conditions such as academic overload, social isolation, and limited access to psychological resources further contribute to student distress. The interplay between these external demands and individual predispositions has been shown to amplify psychological vulnerability, especially during the pandemic recovery phase (Guidotti et al., 2022).

Given this context, the present study seeks to investigate the psychological well-being of university students across two different cultural and academic settings, Italy and the United States. Specifically, the study aims to: (1) compare levels of psychological symptoms, including anxiety, depression, somatization, and hostility, between Italian and American students, and (2) identify key internal predictors, particularly personality traits, which contribute to these symptoms in each group.

By integrating cross-cultural and individual-difference perspectives, this research aims to inform more targeted mental health interventions in university settings and promote the early identification of students at higher psychological risk.

## 2. Materials and Methods

### 2.1. Participants

In this exploratory cross-sectional study, 415 university students (201 Italian and 214 American students), aged between 18 and 45 years, were consecutively recruited. The Italian sample was recruited at the University of Parma (Parma, Emilia-Romagna), while the American sample was collected at Boston College (Chestnut Hill, Massachusetts).

### 2.2. Procedure

In Italy, participants were recruited through posters displayed on campus, which included a link to book an appointment via Outlook calendar. Data were collected in person between April and December 2022. This period corresponded to the full resumption of in-person classes following the COVID-19 pandemic. Before assessment, participants received information about the purpose of the study and gave their informed consent. After completing the questionnaires, they were offered a confidential feedback session to discuss their results.

In the USA, participants were recruited via Boston College’s SONA Systems platform and received 0.5 course credit for participation. The survey was administered online through Qualtrics in anonymous mode between November and December 2022, coinciding with the fall semester exam period.

The survey included six sections: informed consent, demographic information, clinical self-report measures, and a debriefing page. The average completion time was approximately 25 min. For both samples, inclusion criteria were: (1) age ≥ 18 years; (2) provision of informed consent; and (3) no self-reported history of psychiatric or neurological disorders (e.g., head trauma), or physical conditions (e.g., visual or hearing impairments) that might interfere with questionnaire administration.

### 2.3. Measures

The survey consisted of two standardized and validated self-report questionnaires, administered in the following order:

The Symptom Questionnaire (SQ) (Fava et al., 1983; Kellner, 1976) is a self-report instrument designed to assess participants’ well-being and psychological distress during the past week. It includes four subscales derived from factor analysis: Anxiety (A) (23 items); Depression (D) (23 items); Somatization (S) (23 items); and Hostility (H) (23 items). The SQ evaluates both symptoms (i.e., “Tense, tensed up” or “Sad, blue”) and positive feelings (i.e., “Relaxed” or “Feeling friendly”). The weekly version was used. The SQ has demonstrated good psychometric properties in cross-cultural studies. In the present study, Cronbach’s α ranged from 0.83 in the Italian sample to 0.93 in the American sample, indicating excellent internal consistency.

Cattell’s 16 Personality Factor Questionnaire (16PF) (Cattell, 1989; Sirigatti & Stefanile, 2001) comprises 105 items with three response options (true/false/uncertain), designed to assess 16 primary, bipolar, and relatively independent personality dimensions. The factors measured include: A = Warmth (6 items); B = Reasoning (8 items); C = Emotional Stability (6 items); E = Dominance (6 items); F = Liveliness (6 items); G = Rule-Consciousness (6 items); H = Social Boldness (6 items); I = Sensitivity (6 items); L = Vigilance (6 items); M = Abstractedness (6 items); N = Privateness (6 items); O = Apprehension (6 items); Q1 = Openness to Change (6 items); Q2 = Self-Reliance (6 items); Q3 = Perfectionism (6 items); and Q4 = Tension (6 items). An additional scale, the Image Management (IM) scale (7 items), is included to detect the extent to which respondents may attempt to present themselves in a socially desirable manner. A notable feature of the 16PF is its emphasis on respondents’ reactions to concrete situations, rather than relying on abstract self-assessments of personality traits. For instance, items are formulated in terms of specific behaviors, such as: “I enjoy being part of a group” and “I enjoy discussing movies and books with others.” Raw scores are converted into a standardized nine-point scale (ranging from 1 to 9), with scores between 4 and 7 representing the normative range. The instrument has been widely validated in both European and North American populations, supporting its cross-cultural comparability. In this study, Cronbach’s α values for the 16 primary scales ranged from 0.66 to 0.93 in the Italian sample and from 0.68 to 0.87 in the American sample.

### 2.4. Statistical Analysis

All analyses were conducted using SPSS (v28.0.1.0; IBM Corp., Armonk, NY, USA). Descriptive statistics were calculated for demographic (gender, age, nationality) and clinical variables (SQ and 16PF scores), including means (Ms) and standard deviations (SDs). Tests of skewness and kurtosis confirmed the normality of variable distributions. A multicollinearity check revealed no correlation coefficients ≥0.80 among independent variables; variance inflation factors (VIFs) were ≤10 and tolerances ≥0.10, indicating an absence of multicollinearity.

Group comparisons between Italian and American students on socio-demographic variables were conducted as follows: (1) chi-square tests were used to compare gender distributions; (2) independent-samples t-tests were used to compare age. Similarly, differences in clinical variables (SQ and 16PF scores) between the two groups were assessed using additional independent-samples t-tests.

Subsequently, a series of hierarchical linear multiple regression analyses was conducted separately within each national group (Italian and American). For each analysis, one psychological symptom (i.e., anxiety, depression, somatization, or hostility) from the SQ was treated as the dependent variable. Step 1 included gender and age as covariates, and Step 2 included all 16PF personality traits as independent variables. This approach enabled the identification of specific personality predictors of psychological distress within each cultural context.

## 3. Results

Regarding the socio-demographic variables, the American sample was better balanced by gender, while the Italian sample was characterized by a higher proportion of females. Specifically, chi-square tests showed a significant difference in gender distribution between the two groups. Furthermore, independent samples *t*-tests revealed that American students were significantly younger than Italian students (Table 1).

Comparisons between the two groups on psychological symptoms, as measured by the SQ, were conducted using independent samples *t*-tests. Significant differences emerged in the depression and hostility scales, with American students reporting higher levels of both symptoms compared to Italian students. However, descriptively, both groups scored above the clinical cut-off on all four SQ clinical scales.

Looking at personality factors, significant differences emerged when comparing the two samples. American students reported greater sensitivity to emotional arousal (factor H) with lower emotional management skills (factor C). Also, concerning impulse control (Q3) and perseverance (G factor), the scores of Italian students were higher. Furthermore, Italian students reported a greater willingness to share internal states (factor A) and self-opening (factor N) as well as to change in general (factor Q1). Italian students also reported higher levels of liveliness and reasoning ability. On the other hand, they also complained of greater levels of vigilance (factor L) and sensitivity in interpersonal relationships (factor I).

Hierarchical linear multiple regression analyses were conducted separately within the Italian and American samples on the SQ clinical scales where group differences were significant. For depression, Step 1 included gender and age as covariates, and Step 2 included nationality (only in combined analyses). In the combined sample, being female (Gender: B = −1.30; SE = 0.57; *p* < 0.05) and American (Nationality: B = 1.64; SE = 0.64; *p* < 0.01) were significant predictors of higher depression scores. Age was not significant (B = 0.04; SE = 0.08; *p* = n.s.). The model explained 2% of the variance (F = 3.64; *p* < 0.05).

For hostility, a similar regression showed that nationality predicted higher hostility scores (B = 1.36; SE = 0.60; *p* < 0.05) in the combined sample, whereas gender (B = −0.05; SE = 0.53; *p* = n.s.) and age (B = −0.06; SE = 0.08; *p* = n.s.) were not significant predictors. The model explained 3% of variance (F = 3.60; *p* < 0.05).

Separate models for anxiety and somatization revealed that gender was a significant predictor for both symptoms (anxiety: B = −2.11; SE = 0.52; *p* < 0.001; somatization: B = −3.33; SE = 0.57; *p* < 0.001), but nationality and age were not significant predictors in these models (anxiety R^2^ = 0.04, F = 7.14, *p* < 0.001; somatization R^2^ = 0.07, F = 12.30, *p* < 0.001).

Next, hierarchical linear regression analyses were run separately within each sample to explore how personality traits predicted psychological symptoms, controlling for gender and age in Step 1, with personality traits entered in Step 2.

Anxiety was predicted in Italian students by low emotional stability, high emotional arousal (Factor H), and social dependence (Factor Q2). In American students, tension (Factor Q4) and low social boldness (Factor H) were significant predictors. Gender and age did not significantly contribute to explained variance (Table 2).

For depression, tension (Factor Q4) was the main predictor in both samples. Additionally, in the Italian group, low emotional stability (Factor C) and low liveliness (Factor F) explained significant variance (Table 3).

Regarding somatization, low emotional maturity predicted symptoms in both samples. In Americans, nervous tension (Factor Q4) was significant, whereas in Italians, vigilance (Factor L) and apprehension (Factor O) were positively associated with somatic complaints (Table 4).

For hostility, higher anxious arousal poorly regulated by emotional stability predicted symptoms in both groups. Italian students showed greater hostility if characterized by social detachment (low Factor A) and low openness to change (Factor Q1). American students’ hostility was predicted by lower reasoning (Factor B), lower adaptability (Factor Q1), and higher prudence and social reserve (Factors Q3 and N) (Table 5).

## 4. Discussion

This study aimed to investigate the psychological well-being of Italian and American students, comparing samples from two distinct educational and cultural contexts. More specifically, a group of Italian university students was compared to a sample of American college students. The age difference between the two groups was expected and attributed to the distinct educational systems of the two countries (Lyrakos, 2012). Regarding the psychological symptoms of anxiety, depression, somatization, and hostility measured with the SQ, elevated levels were observed in both groups, confirming previous research on the mental health challenges faced by students (Garett et al., 2017; Son et al., 2020; Yang et al., 2021). Furthermore, American students reported higher levels of mood disturbances, with significant differences in depression and hostility that remained even when controlling for gender and age.

Despite these differences, sociodemographic variables such as nationality, gender, and age explained only a small portion of the variance (<10%) in psychological symptoms, suggesting that other variables may have played a key role in modulating academic stress. The strongest predictive models included personality traits, confirming prior evidence that certain temperamental dimensions increase vulnerability to psychological distress by influencing individuals’ responses to environmental stressors (Arias et al., 2020). By conducting separate analyses for each nationality, the study identified distinct personality profiles linked to vulnerability within each group.

These results align with recent research underscoring the significant role of personality traits—particularly neuroticism, low emotional regulation, and low self-efficacy—in shaping mental health trajectories among students (Guidotti et al., 2024a, 2024b). Neurotic traits, in particular, have been consistently linked to higher levels of anxiety, depression, and somatic complaints in young adults navigating stressful academic environments (Dusselier et al., 2005).

For anxiety, the Q4 factor (tension) emerged as a significant predictor in both groups. Higher nervous tension was consistently associated with greater anxiety. However, American students also showed low social boldness, whereas Italian students displayed low emotional stability and high social dependence, suggesting difficulties in emotional autonomy and heightened sensitivity to social evaluation. These traits may reflect anxiety rooted in social approval and attachment dynamics. Trait anxiety and discomfort in social contexts were common features underlying anxiety symptoms in both groups.

Depression was also strongly predicted by nervous tension in both samples. Among Italian students, low emotional stability and reduced liveliness further contributed to depressive symptoms, aligning with the literature that identifies depressive temperament as a risk factor for depression onset (Carrozzino et al., 2019). This association was not found in the American sample, possibly due to cultural or contextual influences.

Somatization was again linked to tension. In American students, somatic symptoms correlated with female gender, high trait anxiety, and low emotional maturity. In contrast, Italian students’ somatization was associated with low emotional regulation, vigilance, and apprehension—traits indicative of a hypervigilant and mistrustful interpersonal style, particularly among women.

As for hostility, both groups shared a pattern of high tension and low emotional stability. However, additional predictors varied: Italian students showed increased hostility when exhibiting social withdrawal, reduced openness, and resistance to change, while American students’ hostility was linked to lower reasoning ability and limited self-disclosure. These findings may reflect culturally influenced models of emotional regulation and interpersonal conflict.

In summary, gender emerged as a consistent predictor of anxiety, depression, and somatization, while nationality significantly predicted mood disturbances, particularly depression and hostility. Age did not appear to be a relevant factor, in contrast to some earlier studies (Saleh et al., 2017; Sussman & Arnett, 2014). This result may be explained by the relatively narrow age range of the participants, who were primarily young adults enrolled in undergraduate programs. The developmental similarities across this stage of emerging adulthood might have attenuated any potential differences in psychological symptoms attributable to age. Furthermore, academic stressors, such as exams and career uncertainty, are likely to affect students similarly regardless of minor age differences within the university context. Still, nationality, gender, and age explained a relatively small share of the variance, indicating that individual-level psychological variables, particularly personality traits, had a stronger impact. Across both groups, low emotional stability and interpersonal sensitivity were robust predictors of mental suffering—echoing longstanding psychological models that highlight neuroticism and introversion as major risk factors for psychopathology (Arias et al., 2020; Ormel et al., 2012; Sandi & Haller, 2015).

These findings support the idea that the pandemic intensified psychological symptoms primarily among individuals predisposed by temperament or personality (Benedetto et al., 2022; Leal Filho et al., 2021). Recent studies confirm that students with higher emotional vulnerability or lower coping flexibility were more likely to experience long-term emotional dysregulation post-COVID (Guidotti et al., 2022). These data may reflect the reduced opportunity for social interactions during lockdowns, which limited the buffering effect of sociality. Such results highlight the importance of preventive strategies targeting emotional self-regulation in at-risk student populations.

According to Porges’ Polyvagal Theory, interpersonal relationships modulate physiological responses to stress by promoting a sense of safety that deactivates defensive systems and enables social engagement (Porges, 2009, 2021). When this safety is disrupted, the nervous system may shift toward states of hypervigilance and avoidance, impeding emotional connection. Therefore, the decrease in social contact during the pandemic may have weakened social buffering and intensified fear of judgment and isolation (Schmidt & Mallott, 2006).

In line with this, the social buffering model of stress also highlights how emotional support can reduce hypothalamic–pituitary–adrenal (HPA) axis activity, lowering cortisol and dampening physiological stress responses (Eisenberger & Cole, 2012; Gunnar & Hostinar, 2015). This model is supported by organizational research showing that social support moderates the impact of workload on perceived stress, both psychologically and biologically (Karasek & Theorell, 1990). These perspectives converge with interpersonal neuroscience, which emphasizes that the brain is evolutionarily wired for connection and that healthy development depends on relational safety and attunement (Cozolino, 2014). Taken together, these theories suggest that both personal and professional relationships play a central role in psychophysiological resilience, especially under stress.

While the results of this study are promising, future research is needed to better understand the vulnerability of college and university students to psychological distress. Larger, more diverse samples, including students from multiple regions or countries, would strengthen generalizability. Several limitations should be acknowledged. First, both samples had a gender imbalance, especially the Italian group (74% female). Second, data collection occurred at different time points: Italian data were collected from April to December 2022, whereas American data were gathered between November and December 2022. Although both periods coincided with exam seasons, contextual stressors such as the lingering impact of COVID-19 restrictions may have varied, especially in Italy before April. Third, the recruitment methods differed. Although the same instruments were used in both countries, the difference in survey administration methods (in-person vs. online) may have influenced response styles, particularly for sensitive items, such as psychological symptoms and socially desirable traits. This potential bias is acknowledged as a methodological limitation. Nonetheless, it is important to note that validity scores from the personality questionnaire were low in both groups, suggesting honest responses. Additionally, both questionnaires used in the present study demonstrated evidence of cross-cultural applicability, although with important considerations (Habibi et al., 2025). The 16PF has been validated in several international contexts, including Israel, the United Kingdom, and South Africa, showing largely comparable psychometric properties and stable factor structures, especially for the primary scales (van Eeden & Prinsloo, 1997; Zak, 1976). Only differences in second-order factors (which were not used in this study) across cultures (e.g., between Japanese and Western samples) highlighted the need for caution in interpretation (Golden, 1978). Similarly, the SQ has undergone cross-cultural validation, particularly in its Italian translation, which demonstrated good reliability and sensitivity in assessing psychological distress in both clinical and non-clinical populations (Fava et al., 1983). Overall, these results are consistent with previous validation research and support the use of both instruments in cross-cultural comparisons, including among Italian and American student populations.

Despite these limitations, this study contributes to a growing body of evidence showing that university students are at risk for elevated psychological symptoms. More research is needed to clarify how internal (e.g., personality traits and coping styles) and external (e.g., social support, academic stress) stressors interact to influence mental health. The literature consistently proved that high academic stress increases psychological symptoms, while strong social support and adaptive coping strategies can mitigate these effects (Dong et al., 2024; Huang et al., 2021; McLean et al., 2022). Although not assessed in the present study, these factors should be considered in future research to better capture the complexity of students’ psychological well-being. Future research should also explore how vulnerable personality traits are expressed and interact with stress in different cultural contexts. In this regard, it is important to highlight that this study had an observational and exploratory nature. As such, we focused primarily on the main effects of demographic and personality variables on psychological symptoms, without including interaction terms between individual traits and cultural variables or other psychological variables. Future studies may benefit from exploring the moderating role of cultural context in the association between personality and psychological distress. For instance, moderation models could help determine whether specific traits (i.e., emotional stability or conscientiousness) have differential impacts on mental health depending on national or institutional contexts. Such analyses would offer a more nuanced understanding of intercultural dynamics and could support the development of more tailored and culturally sensitive interventions.

A transcultural perspective could indeed offer useful insights for adapting early interventions and promoting culturally sensitive strategies for promoting resilience and psychosocial well-being.

This type of research should be strongly encouraged, especially in light of the growing consensus on the importance of tailoring interventions to individual temperamental profiles. Targeting emotional dysregulation, social inhibition, and cognitive vulnerability may improve the effectiveness of mental health programs in higher education (Guidotti et al., 2024a, 2024b). For example, identifying key personality predictors, such as emotional instability, nervous tension, and social detachment, offers practical avenues for targeted mental health interventions. In this regard, universities should consider implementing early screening programs focused on these personality traits to identify students at higher risk for psychological distress. Such screenings could be integrated into routine student health assessments, allowing for timely referral to counseling and support services. Furthermore, preventive interventions should focus on developing students’ emotional regulation and social engagement skills. Individualized workshops, psychoeducational programs, and peer support groups could be effective in promoting resilience, especially for students who exhibit social isolation or resistance to change. Notwithstanding, teachers and counselors should be able to recognize behavioral cues related to these personality profiles and facilitate access to mental health resources. Future research should extend these efforts to diverse populations and explore the impact of culturally adapted interventions.

## 5. Conclusions

This study confirmed that university and college students are highly vulnerable to psychological distress, with symptoms of anxiety, depression, somatization, and hostility emerging across both Italian and American samples. Although socio-demographic variables (i.e., gender and nationality) were partially associated with symptom levels, their explanatory power was limited. Instead, personality traits, particularly low emotional regulation, high tension, and interpersonal sensitivity, emerged as stronger and more consistent predictors of psychological symptoms across cultural contexts. These findings suggest that the emotional and relational profiles of students are more critical to their mental health than external factors, such as nationality or age. This calls for a shift in how university mental health services conceptualize and address student vulnerability.

Given that personality dimensions such as trait anxiety, poor emotional regulation, and social withdrawal significantly predicted mental health outcomes, mental health services in academic settings should consider integrating personality assessment into routine student care. Specifically, early identification of high-risk profiles can inform targeted psychoeducational or therapeutic interventions. Universities should also strengthen preventive mental health strategies, such as: (1) screening for psychological vulnerability during intake processes; (2) delivering workshops on emotional self-regulation, interpersonal skills, and stress management; (3) promoting peer mentoring and structured social support programs to buffer stress and isolation; (4) encouraging culturally sensitive approaches to counseling that take into account transcultural differences in personality and symptom expression. Finally, this study reinforces the idea that resilience is not merely situational but also dispositional, shaped by enduring personality traits. By recognizing and addressing these internal risk factors, universities can better support students’ emotional well-being and academic performance.

## Figures and Tables

**Table 1:** Comparisons of socio-demographic features between Italian and American students.

Variable	Italian Group (n = 201)	American Group (n = 214)	Total Sample (n = 415)	*t* or χ^2^	*p*
Age, M (SD)	23.48 (4.69)	19.42 (1.02)	21.37 (3.90)	*t* (414) = −4.11	<0.001
Gender, N (%)				χ^2^ (1, *N* = 414) = 16.29	<0.001
Male	52 (25.87%)	96 (44.86%)	146 (35.18%)		
Female	149 (74.13%)	118 (55.14%)	267 (64.34%)		
Symptom Questionnaire, M (SD)					
Anxiety	9.93 (4.83)	10.79 (5.22)	10.37 (5.05)	*t* (414) = −1.93	n.s.
Depression	7.76 (5.10)	9.04 (5.60)	8.42 (5.39)	*t* (414) = −2.44	<0.05
Somatization	9.84 (5.70)	10.39 (5.61)	10.13 (5.65)	*t* (414) = −0.99	n.s.
Hostility	5.99 (4.72)	7.40 (5.27)	6.79 (5.07)	*t* (414) = −3.15	<0.01
16-Personality Factors Questionnaire, M (SD)					
Image Management (IM)	5.65 (2.36)	5.10 (1.90)	5.37 (2.15)	*t* (414) = 2.62	<0.01
Warmth (A)	6.02 (2.61)	5.51 (2.30)	5.76 (2.46)	*t* (414) = 2.12	<0.05
Reasoning (B)	4.96 (1.39)	3.93 (1.35)	4.43 (1.46)	*t* (414) = 7.71	<0.001
Emotional Stability (C)	6.53 (2.46)	5.87 (2.16)	6.19 (2.33)	*t* (414) = 2.93	<0.01
Dominance (E)	5.56 (2.00)	5.37 (1.90)	5.47 (1.95)	*t* (414) = 0.98	n.s.
Liveliness (F)	7.16 (2.49)	6.04 (1.98)	6.58 (2.30)	*t* (414) = 5.10	<0.001
Rule-consciousness (G)	7.19 (2.00)	5.33 (2.02)	6.23 (2.22)	*t* (414) = 9.42	<0.001
Social Boldness (H)	6.07 (2.34)	6.52 (2.02)	6.31 (2.19)	*t* (414) = −2.10	<0.05
Sensitivity (I)	6.31 (2.09)	5.57 (2.04)	5.93 (2.10)	*t* (414) = 3.71	<0.001
Vigilance (L)	7.30 (2.23)	6.11 (1.94)	6.69 (2.17)	*t* (414) = 5.79	<0.001
Abstractness (M)	7.55 (1.85)	7.42 (1.87)	7.48 (1.86)	*t* (414) = 0.69	n.s.
Privateness (N)	6.35 (1.96)	6.75 (1.79)	6.56 (1.88)	*t* (414) = −2.17	<0.05
Apprehension (O)	6.04 (2.06)	6.10 (1.82)	6.07 (1.94)	*t* (414) = −0.36	n.s.
Openness to Change (Q1)	6.01 (2.04)	4.68 (2.09)	5.32 (2.17)	*t* (414) = 6.56	<0.001
Self-Reliance (Q2)	6.22 (1.92)	6.27 (1.81)	6.25 (1.86)	*t* (414) = −0.28	n.s.
Perfectionism (Q3)	6.93 (2.31)	6.22 (2.07)	6.57 (2.22)	*t* (414) = 3.28	<0.001
Tension (Q4)	6.24 (2.44)	6.34 (2.45)	6.30 (2.44)	*t* (414) = −0.51	n.s.

Legend: n = number of subjects; M = mean; n.s. = non significant; SD = standard deviation.

**Table 2:** Hierarchical linear multiple regression analyses of personality traits and control variables on anxiety (total score) for Italian (n = 201) and American (n = 214) samples.

	Italian						American				
	*b*	SE	*β*	*t*	*p*		*b*	SE	*β*	*t*	*p*
Step 1											
Gender	−1.17	0.72	−0.11	−1.62	n.s.	Gender	−0.83	0.58	−0.08	−1.43	n.s.
Age	−0.10	0.07	−0.10	−1.49	n.s.	Age	0.18	0.28	0.04	0.64	n.s.
Step 2											
16PF Factor C	−0.53	0.13	−0.27	−3.95	<0.001	16PF Factor Q4	1.22	0.12	0.57	10.18	<0.001
16PF Factor Q4	0.46	0.14	0.23	3.38	<0.001	16PF Factor H	−0.28	0.15	−0.11	−1.96	<0.05
16PF Factor Q2	−0.34	0.16	−0.14	−2.10	<0.05						
	R^2^ = 0.21 *** (F = 11.67)						R^2^ = 0.39 *** (F = 35.02)				

Note: All predictor variables are mean-centered; gender was coded as female = 0 and male = 1. Legend: *** = *p* < 0.001; b = unstandardized regression coefficient; β = standardized regression coefficient; n.s. = non significant; *p* = *p* value; SE = standard error; *t* = *t* test.

**Table 3:** Hierarchical linear multiple regression analyses of personality traits and control variables on depression (total score) for Italian (n = 201) and American (n = 214) samples.

	Italian						American				
	*b*	SE	*β*	*t*	*p*		*b*	SE	*β*	*t*	*p*
Step 1											
Gender	−0.52	0.72	−0.04	−0.72	n.s.	Gender	−0.11	0.69	−0.01	−0.16	n.s.
Age	−0.02	0.07	−0.02	−0.25	n.s.	Age	0.20	0.33	0.04	0.62	n.s.
Step 2											
16PF Factor Q4	0.68	0.14	0.32	4.79	<0.001	16PF Factor Q4	1.15	0.14	0.51	8.28	<0.001
16PF Factor C	−0.48	0.13	−0.23	−3.55	<0.001						
16PF Factor F	−0.39	0.13	−0.19	−2.97	<0.01						
	R^2^ = 0.28 *** (F = 16.28)						R^2^ = 0.25 *** (F = 24.87)				

Note: All predictor variables are mean-centered; gender was coded as female = 0 and male = 1. Legend: *** = *p* < 0.001; b = unstandardized regression coefficient; β = standardized regression coefficient; n.s. = non significant; *p* = *p* value; SE = standard error; *t* = *t* test.

**Table 4:** Hierarchical linear multiple regression analyses of personality traits and control variables on somatization (total score) for Italian (n = 201) and American (n = 214) samples.

	Italian						American				
	*b*	SE	*β*	*t*	*p*		*b*	SE	*β*	*t*	*p*
Step 1											
Gender	−1.74	0.84	−0.13	−2.08	<0.05	Gender	−2.80	0.65	−0.25	−4.31	<0.001
Age	−0.11	0.08	−0.09	−1.36	n.s.	Age	0.28	0.31	0.05	0.91	n.s.
Step 2											
16PF Factor C	−0.56	0.16	−0.24	−3.56	<0.001	16PF Factor Q4	0.90	0.14	0.39	6.51	<0.001
16PF Factor L	0.56	0.17	0.22	3.23	<0.001	16PF Factor C	−0.47	0.15	−0.18	−3.08	<0.01
16PF Factor O	0.53	0.18	0.19	2.95	<0.01						
	R^2^ = 0.23 *** (F = 12.44)						R^2^ = 0.34 *** (F = 28.36)				

Note: All predictor variables are mean-centered; gender was coded as female = 0 and male = 1. Legend: *** = *p* < 0.001; b = unstandardized regression coefficient; β = standardized regression coefficient; n.s. = non significant; *p* = *p* value; SE = standard error; *t* = *t* test.

**Table 5:** Hierarchical linear multiple regression analyses of personality traits and control variables on hostility (total score) for Italian (n = 201) and American (n = 214) samples.

	Italian						American				
	*b*	SE	*β*	*t*	*p*		*b*	SE	*β*	*t*	*p*
Step 1											
Gender	0.30	0.70	0.03	0.43	n.s.	Gender	0.91	0.67	0.09	1.36	n.s.
Age	−0.04	0.07	−0.04	−0.61	n.s.	Age	−0.29	0.32	−0.06	−0.89	n.s.
Step 2											
16PF Factor C	−0.50	0.13	−0.26	−3.79	<0.001	16PF Factor C	−0.89	0.16	−0.36	−5.70	<0.001
16PF Factor Q4	0.33	0.14	0.17	2.48	<0.05	16PF Factor Q4	0.46	0.14	0.22	3.28	<0.001
16PF Factor A	−0.37	0.12	−0.21	−3.13	<0.01	16PF Factor B	−0.53	0.24	−0.13	−2.20	<0.05
16PF Factor Q1	−0.36	0.15	−0.15	−2.35	<0.05	16PF Factor N	0.41	0.18	0.14	2.23	<0.05
	R^2^ = 0.21 *** (F = 9.49)						R^2^ = 0.23 *** (F = 11.79)				

Note: All predictor variables are mean-centered; gender was coded as female = 0 and male = 1. Legend: *** = *p* < 0.001; b = unstandardized regression coefficient; β = standardized regression coefficient; n.s. = non significant; *p* = *p* value; SE = standard error; *t* = *t* test.

## Data Availability

The data presented in this study are available upon reasonable request from the corresponding author.
